# Predicting breast cancer types on and beyond molecular level in a multi-modal fashion

**DOI:** 10.1038/s41523-023-00517-2

**Published:** 2023-03-22

**Authors:** Tianyu Zhang, Tao Tan, Luyi Han, Linda Appelman, Jeroen Veltman, Ronni Wessels, Katya M. Duvivier, Claudette Loo, Yuan Gao, Xin Wang, Hugo M. Horlings, Regina G. H. Beets-Tan, Ritse M. Mann

**Affiliations:** 1 Department of Radiology, Netherlands Cancer Institute (NKI), Plesmanlaan 121, 1066 CX Amsterdam, The Netherlands; 2 GROW School for Oncology and Development Biology, Maastricht University, P. O. Box 616, 6200 MD Maastricht, The Netherlands; 3 Department of Diagnostic Imaging, Radboud University Medical Center, Geert Grooteplein Zuid 10, 6525 GA Nijmegen, The Netherlands; 4 Faculty of Applied Sciences, Macao Polytechnic University, 999078 Macao SAR, China; 5 Department of Radiology, Hospital Group Twente (ZGT), Almelo, The Netherlands; 6 Multi-Modality Medical Imaging Group, TechMed Centre, University of Twente, Enschede, The Netherlands; 7 Department of Radiology, Haga Teaching Hospital, The Hague, The Netherlands; 8 Department of Radiology and Nuclear Medicine, Cancer Center Amsterdam, Amsterdam UMC, Vrije Universiteit Amsterdam, Amsterdam, The Netherlands; 9 Division of Pathology, Netherlands Cancer Institute (NKI), Plesmanlaan 121, 1066 CX Amsterdam, The Netherlands

**Keywords:** Breast cancer, Breast cancer

## Abstract

Accurately determining the molecular subtypes of breast cancer is important for the prognosis of breast cancer patients and can guide treatment selection. In this study, we develop a deep learning-based model for predicting the molecular subtypes of breast cancer directly from the diagnostic mammography and ultrasound images. Multi-modal deep learning with intra- and inter-modality attention modules (MDL-IIA) is proposed to extract important relations between mammography and ultrasound for this task. MDL-IIA leads to the best diagnostic performance compared to other cohort models in predicting 4-category molecular subtypes with Matthews correlation coefficient (MCC) of 0.837 (95% confidence interval [CI]: 0.803, 0.870). The MDL-IIA model can also discriminate between Luminal and Non-Luminal disease with an area under the receiver operating characteristic curve of 0.929 (95% CI: 0.903, 0.951). These results significantly outperform clinicians’ predictions based on radiographic imaging. Beyond molecular-level test, based on gene-level ground truth, our method can bypass the inherent uncertainty from immunohistochemistry test. This work thus provides a noninvasive method to predict the molecular subtypes of breast cancer, potentially guiding treatment selection for breast cancer patients and providing decision support for clinicians.

## Introduction

Breast cancer is the most common malignant tumor in women, and it has a high degree of heterogeneity in terms of clinicopathological characteristics, prognosis, and response to treatment^1–5^. Breast cancers can be divided into molecular subtypes, which is based upon the genetic profile of the cancers, but in practice is usually based upon the expression levels of the estrogen receptor (ER), progesterone receptor (PR), human epidermal growth factor receptor 2 (HER2) and Ki-67. The resulting molecular subtypes are known as Luminal A (ER+ and/or PR+, HER2−, low Ki-67), Luminal B (ER+ and/or PR+, HER2− with high Ki-67 or HER2+ with any Ki-67 status), HER2-enriched (ER−, PR−, HER2+) and Triple-negative (TN) breast cancer (ER−, PR−, HER2−)^6^. These molecular subtypes are an important prognostic factor and can guide pre- and postoperative systemic therapy, because these therapies typically target these receptors^7,8^. In generally, Luminal A breast cancer has the best prognosis and is usually treated with endocrine therapy; Luminal B has a good prognosis and can be treated with endocrine therapy, cytotoxic chemotherapy or targeted therapy; HER2-enriched cancers are nowadays treated with targeted therapy in combination with cytotoxic chemotherapy, which has strongly improved the prognosis; TN breast cancer still has the worst prognosis and cytotoxic (neoadjuvant) chemotherapy is the main treatment option^9–13^. At present, the molecular subtypes of breast cancer are determined by immunohistochemistry (IHC) analysis of biopsy specimen, which is a surrogate for genetic testing, as the genetic analysis is quite costly^14,15^. Unfortunately, the biopsy procedure limits the assessment to a small part of the tumor, which might prevent obtaining a full impression of the nature of the lesion. Differentiation within breast cancer may lead to subclones with different receptor expression, which may not be fully captured by analysis of core biopsies. In addition, in particular, if the HER2 result on IHC test is score 2+ (meaning borderline), genetic test method, such as fluorescence in situ hybridization, have to be used to retest the tissue to ensure the result is accurate, thus increasing the cost and taking longer to return result^16–18^. Neither IHC nor in situ hybridization is available everywhere around the world^19^, which may lead to substantial under- or overtreatment of patients. For example, lack of adequate receptor staining may lead to giving drugs that don’t work (e.g. tamoxifen in an ER- patient), or omission of drugs with a very strong effect in specific patients (e.g. trastuzumab in Her2+ disease). Therefore, there is a need for an effective technique to assist in the analysis of the entire breast lesions to accurately predict the molecular subtypes of breast cancer and provide decision support.

With the continuous development of artificial intelligence (AI), AI-based methods have been widely used in the field of breast imaging mainly for the evaluation of screening mammograms, segmentation of breast tumors and some other classification tasks^20–27^. Recent studies have shown that the performance of deep learning is close to the average level of radiologists in the evaluation of screening mammograms^23,28^. Some studies have shown that AI-based method can predict molecular subtypes of breast cancer from hematoxylin-eosin-stained breast cancer pathological images^29–31^. However, breast tumors are highly heterogeneous, and the results of tissue samples taken from a specific location in the breast tumor may not be representative of the entire tumor. In some studies, a combination of medical imaging and AI has been used to predict the molecular subtypes of breast cancer, however, most research efforts are based on traditional machine learning methods and provide limited accuracy cancer^32–35^. In addition, these previous studies only analyzed single-modality images, such as mammography, ultrasound or MRI and did not integrate features from different imaging modalities. Most studies are performed using breast MRI, but this examination is generally not yet available at the time of cancer detection. Moreover, obtaining breast MRI is not standard for all breast cancer patients in most countries, and is not uniformly done even within Europe^36^.

Mammography (MG) and ultrasound (US) are routinely used during breast cancer screening, and are commonly used to identify, and characterize breast lesions and guide biopsy. Different than for breast MRI, these two modalities are virtually always and everywhere available at the time of cancer diagnosis. In this work, we thus investigate the feasibility of predicting molecular subtypes of breast cancer from the combination of MG and US using a multi-modal deep learning model incorporating the attention mechanism. Moreover, we conduct a reader study, comparing our multi-modal AI prediction with the prediction by experienced clinicians on the radiological images. We also assess whether AI could be an alternative to a gene-test in determining the class for borderline cases with a HER2 score of 2+. The Matthews correlation coefficient (MCC) of our multi-modal AI in predicting 4-category molecular subtypes is 0.837 (95% confidence interval [CI]: 0.803, 0.870), and the area under the receiver operating characteristic curve (AUC) for distinguishing Luminal and Non-Luminal diseases is 0.929 (95% CI: 0.903, 0.951). This work thus provides a noninvasive method to predict the molecular subtypes of breast cancer, potentially guiding treatment selection for breast cancer patients.

## Results

### Characteristics of cases

**Fig. 1:**
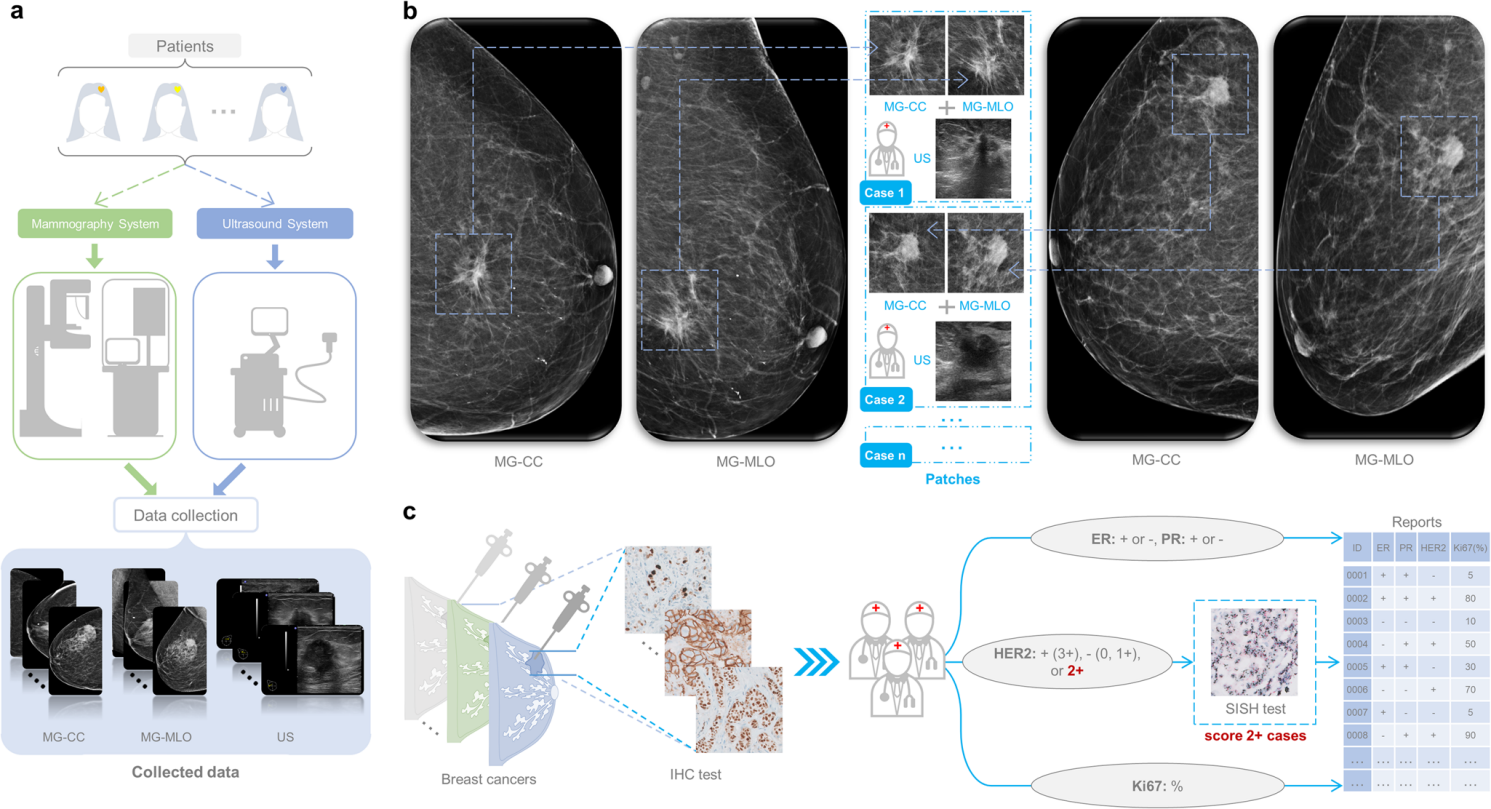
Workflow diagrams. **a** Data collection for mammography and ultrasound. **b** Examples of extracting patches for the lesion locations by the dedicated breast radiologists. **c** Test procedure for expression levels of all indicators. MG mammography, CC craniocaudal, MLO mediolateral oblique, US ultrasound, IHC immunohistochemistry, ER estrogen receptor, PR progesterone receptor, HER2 human epidermal growth factor receptor 2, SISH silver-enhanced in situ hybridization.

Between January 2010 to November 2019, a total of 3360 paired cases (MG and corresponding US) were obtained (as shown in Fig. 1a, b), and these cancer cases were grouped into 4 molecular subtypes based on the information of ER, PR, HER2 and Ki67 from IHC findings and silver-enhanced in situ hybridization (SISH) test (as shown in Fig. 1c). In total, we included 1494 cases of Luminal A, 905 cases of Luminal B, 386 cases of HER2-enriched, and 575 cases of TN breast cancer. The characteristics of breast cancer cases used in this study are summarized in Table 1. There are no significant differences between the tumor characteristics of training and test cohort (all *p* values > 0.05).

**Table 1:** Characteristics of breast cancer cases in this study.

	All	Training cohort	Test cohort	*p* value	Observer study cohort
Number	3360	2688 (80.0%)	672 (20.0%)	–	168 (5.0%)
Age	54 ± 9	54 ± 8	54 ± 10	0.312	54 ± 12
Tumor size (mm)	19.9 ± 10.4	20.0 ± 10.5	19.5 ± 9.9	0.285	19.2 ± 9.4
Grade				0.057	
1	466	370 (13.8%)	96 (14.3%)	–	30 (17.9%)
2	1762	1386 (51.6%)	376 (56.0%)	–	82 (48.8%)
3	1132	932 (34.7%)	200 (29.8%)	–	56 (33.3%)
Density category in MG				0.739	
A	411	336 (12.5%)	75 (11.2%)	–	24 (14.3%)
B	1434	1132 (42.1%)	302 (44.9%)	–	83 (49.4%)
C	1314	1054 (39.2%)	260 (38.7%)	–	55 (32.7%)
D	201	166 (6.2%)	35 (5.2%)	–	6 (3.6%)
BI-RADS category				0.313	
4 (4a, 4b, 4c)	2052	1653 (61.5%)	399 (59.4%)	–	89 (53.0%)
5	1308	1035 (38.5%)	273 (40.6%)	–	79 (47.0%)
Histologic type				0.345	
Invasive ductal carcinoma	2512	2019 (75.1%)	493 (73.4%)	–	118 (70.2%)
Invasive lobular carcinoma	394	312 (11.6%)	82 (12.2%)	–	26 (15.5%)
Others	454	357 (13.3%)	97 (14.4%)	–	24 (14.3%)
Molecular subtypes				0.126	
Luminal A	1494	1214 (45.2%)	280 (41.7%)	–	67 (39.9%)
Luminal B	905	721 (26.8%)	184 (27.4%)	–	49 (29.2%)
HER2-enriched	386	298 (11.1%)	88 (13.1%)	–	23 (13.7%)
Triple-negative	575	455 (16.9%)	120 (17.9%)	–	29 (17.3%)

Values in parentheses are the percentage. Ages and Tumor sizes are reported as mean ± standard deviation. Density category A, the breasts are almost entirely fatty. Density category B, there are scattered areas of fibroglandular density. Density category C, heterogeneously dense. Density category D, extremely dense.

*MG* mammography, *BI-RADS* Breast imaging reporting and data system.

### Base model selection

**Fig. 2:**
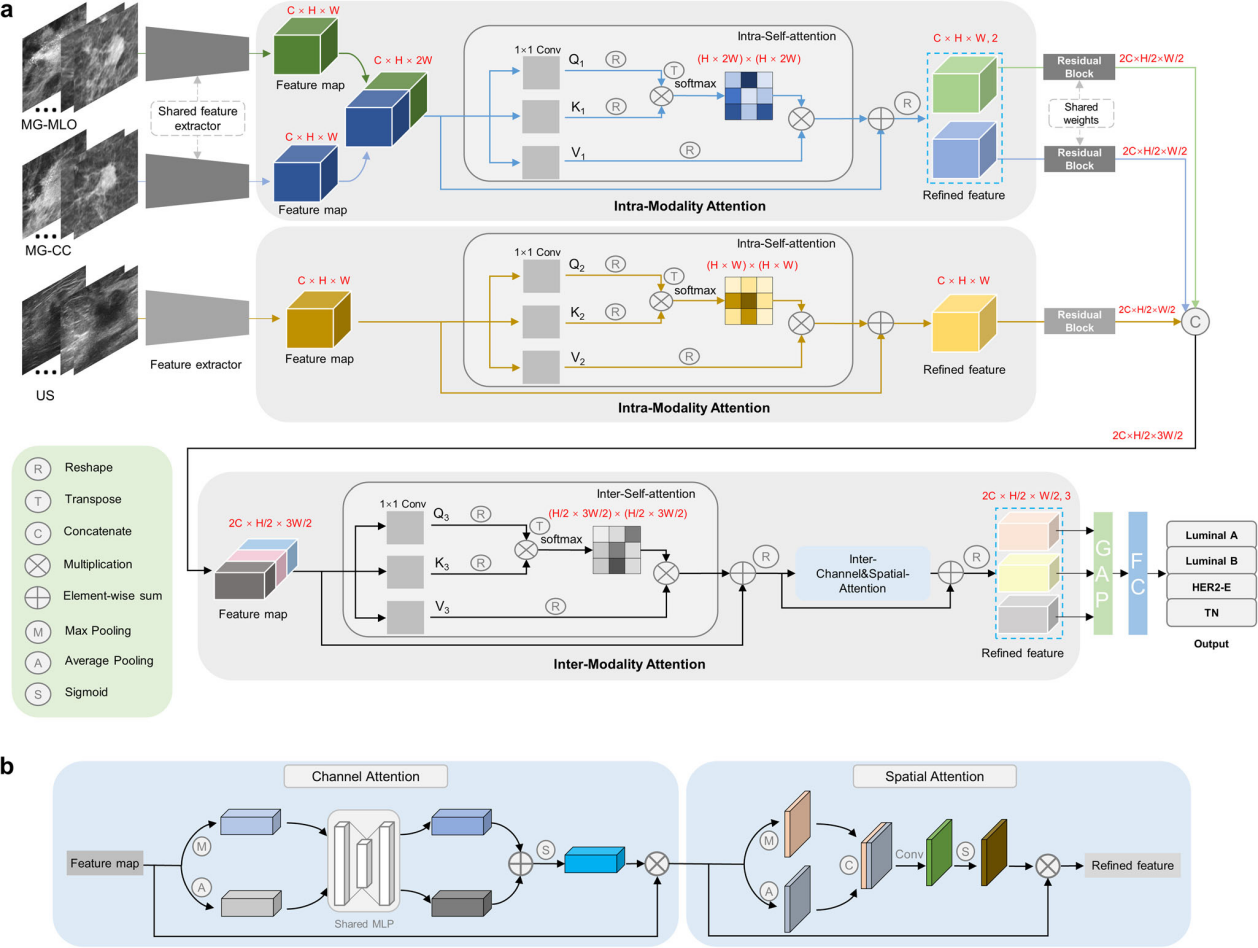
The scheme for this work. **a** The proposed multi-modal deep learning with intra- and inter-modality attention model. **b** The structure of channel and spatial attention. C channel, H height, W width, Q query, K key, V value, MG mammography, US ultrasound, MLO mediolateral oblique view, CC craniocaudal view, GAP global average pooling, FC fully-connected layer, HER2-E HER2-enriched, TN triple-negative.

To find the most suitable base/backbone model for our study, the performance of ResNet34, ResNet50, ResNet101, and Inceptionv3 in predicting molecular subtypes of breast cancer were compared. The ResNet34, ResNet50, ResNet101 were applied, inspired by the work of He et al.^37^, and the Inceptionv3 was applied according to the work of Szegedy et al.^38^. As shown in Table 2 (from line 3 to line 6), ResNet50 showed better performance and was therefore selected as the base model. After selecting the optimal base model, the intra-modality attention module and the inter-modality attention module were incorporated into the model as multi-modal deep learning with intra- and inter-modality attention modules (MDL-IIA) (Fig. 2). At the same time, the channel attention module, Squeeze-and-Excitation^39^ (SE) was also selected as a cohort model for benchmarking.

**Table 2:** Comparison of performance between different models for predicting 4-category molecular subtypes of breast cancer in the test cohort (*n* = 672).

Method	Modality	Accuracy (%)	Precision (%)	Recall (%)	F1-score	MCC
US-ResNet50	US	81.1 [78.0, 83.9]	79.7 [76.3, 82.9]	76.9 [73.3, 80.2]	0.774 [0.739, 0.807]	0.731 [0.689, 0.772]
MG-ResNet50	MG	82.0 [79.0, 84.8]	81.3 [77.8, 84.5]	78.3 [74.9, 81.5]	0.787 [0.753, 0.821]	0.744 [0.704, 0.785]
Multi-ResNet50	MG + US	84.4 [81.5, 87.1]	83.7 [80.4, 86.9]	81.3 [77.8, 84.5]	0.820 [0.786, 0.852]	0.777 [0.736, 0.814]
Multi-ResNet34	MG + US	83.3 [80.4, 86.0]	83.1 [80.0, 86.3]	79.8 [76.5, 83.1]	0.803 [0.769, 0.838]	0.764 [0.724, 0.803]
Multi-ResNet101	MG + US	83.0 [80.1, 85.9]	82.5 [79.1, 85.7]	79.4 [76.1, 82.6]	0.799 [0.766, 0.832]	0.759 [0.720, 0.798]
Multi-Inceptionv3	MG + US	82.6 [79.6, 85.4]	82.0 [78.7, 85.2]	78.9 [75.7, 82.0]	0.794 [0.761, 0.827]	0.753 [0.713, 0.793]
Multi-ResNet34+SE	MG + US	85.1 [82.3, 87.8]	85.1 [82.0, 88.0]	82.1 [78.8, 85.2]	0.826 [0.794, 0.857]	0.790 [0.751, 0.826]
Multi-ResNet50+SE	MG + US	86.0 [83.3, 88.5]	86.0 [82.9, 88.8]	82.5 [79.2, 85.7]	0.835 [0.802, 0.867]	0.801 [0.763, 0.838]
Multi-ResNet101+SE	MG + US	84.8 [82.1, 87.5]	84.4 [81.5, 87.2]	81.6 [78.5, 84.8]	0.822 [0.791, 0.854]	0.784 [0.749, 0.822]
Multi-Inceptionv3+SE	MG + US	83.3 [80.4, 86.2]	82.3 [79.1, 85.6]	79.7 [76.3, 82.9]	0.800 [0.767, 0.833]	0.764 [0.724, 0.802]
Proposed (MDL-IIA)	MG + US	88.5 [86.0, 90.9]	87.8 [85.0, 90.7]	85.4 [82.2, 88.4]	0.862 [0.831, 0.892]	0.837 [0.803, 0.870]

Values in brackets are 95% confidence intervals [95% CI, %].

*MG* mammography, *US* ultrasound, *SE* Squeeze-and-Excitation, *MCC* Matthews correlation coefficient, *MDL-IIA* multi-modal deep learning with intra- and inter-modality attention modules.

### Prediction of 4-category molecular subtypes of breast cancer

The results of different models in terms of the evaluation metrics for predicting 4-category molecular subtypes of breast cancer in the test cohort (*n* = 672) are displayed in Table 2. The Multi-ResNet50 model had an accuracy of 84.4%, precision of 83.7%, recall of 81.3%, F1-score of 0.820, and MCC of 0.777. In contrast, the multi-modal deep learning model Multi-ResNet50 (combining MG and US) led to better MCC of 0.777, which was significantly higher than the MCC of 0.744 (*p* value < 0.001) based on MG images only and the MCC of 0.731 (*p* value < 0.001) based on US images, respectively (line 1 to 3). The lower part of Table 2, shows the comparison of the proposed method based upon the Multi-ResNet50 with intra- and inter-modality attention (MDL-IIA) to other multi-modality network designs. Use of the MDL-IIA model resulted in the best diagnostic performance for predicting 4-category molecular subtypes with an accuracy of 88.5%, which was 2.5% higher than the second-best model (Multi-ResNet50+SE). In particular, there were 112 cases with an IHC test result of 2+ (borderline) for HER2 in the test cohort, and the proposed model achieved an accuracy of 86.6% (97 of 112) for these cases.

**Fig. 3:**
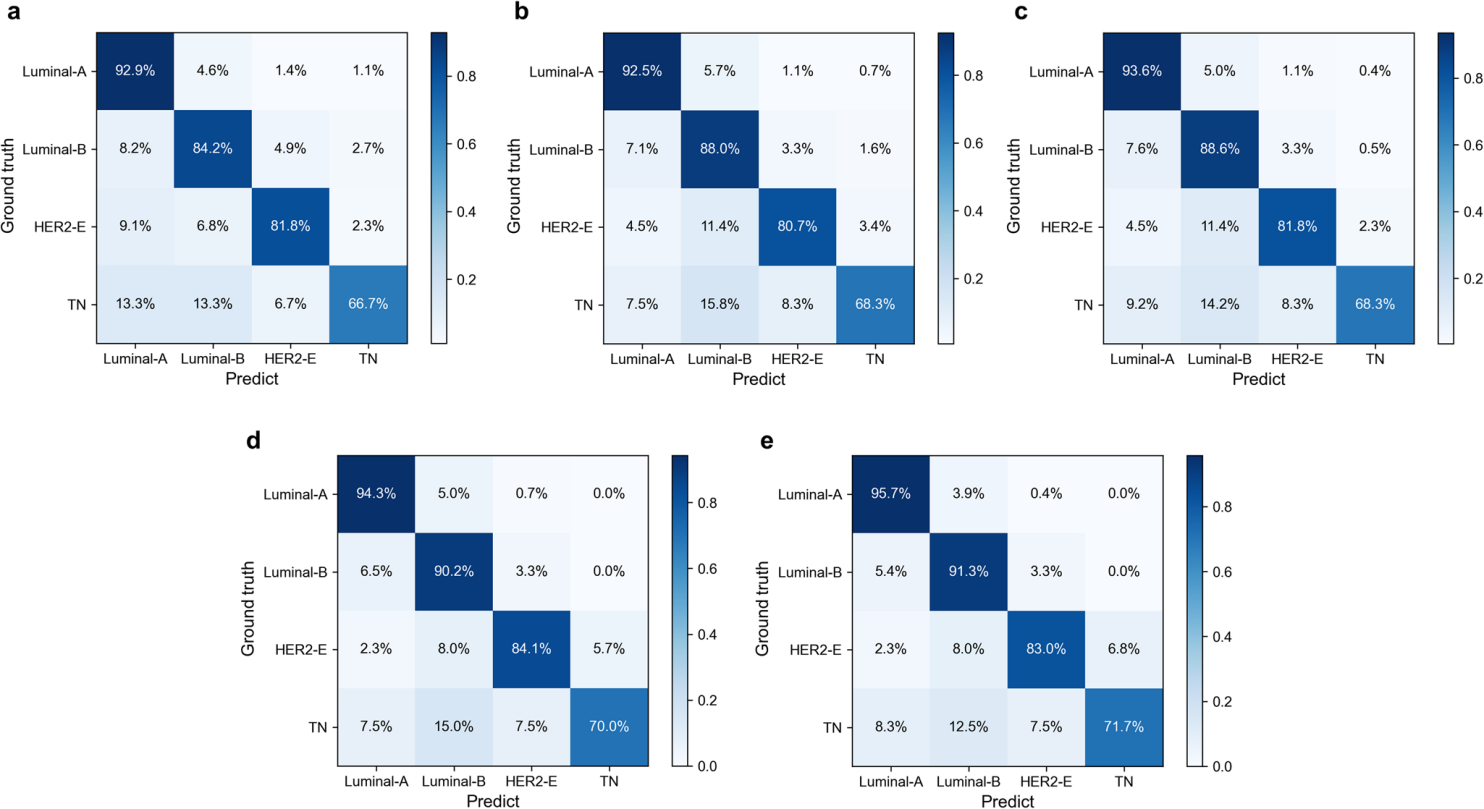
The normalized confusion matrix for the prediction of 4-category molecular subtypes of breast cancer by different models in the test cohort (*n* = 672). **a–e** Multi-ResNet, MulR-interSA, MulR-iiSA, MulR-interCSA, and proposed MDL-IIA models. MulR Multi-ResNet, SA self-attention, iiSA intra- and inter-self-attention, CSA channel and spatial attention, interSA inter self-attention, interCSA inter channel and spatial attention, MDL-IIA multi-modal deep learning with intra- and inter-modality attention modules, HER2-E HER2-enriched, TN triple-negative.

**Fig. 4:**
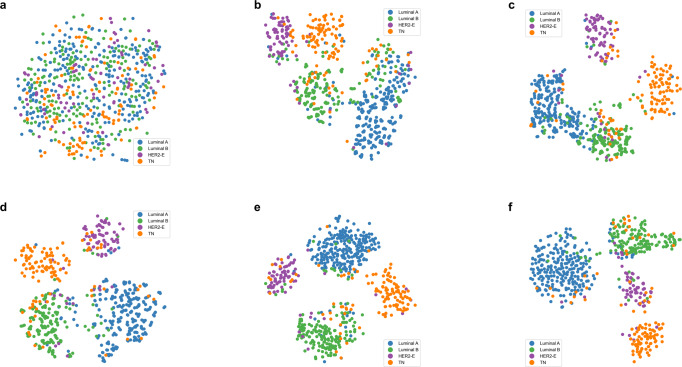
The visualization results of t-SNE for the task of predicting 4-category molecular subtypes of breast cancer in the test cohort (*n* = 672). **a–f** Original test dataset, Multi-ResNet, MulR-interSA, MulR-iiSA, MulR-interCSA, and proposed MDL-IIA models. MulR Multi-ResNet, SA self-attention, iiSA intra- and inter-self-attention, CSA channel and spatial attention, interSA inter self-attention, interCSA inter channel and spatial attention, MDL-IIA multi-modal deep learning with intra- and inter-modality attention modules, HER2-E HER2-enriched, TN triple-negative.

The ablation test results are shown in Table 3. Combining the Multi-ResNet50 model with the attention mechanism in general improves the performance of the model, and the proposed model MDL-IIA has the best performance. Figure 3 shows the normalized confusion matrix for predicting 4-category molecular subtypes of breast cancer. The results indicated that the models recognized Luminal A and Luminal B better than HER2-enriched and TN, and we can observe that the designed attention module can potentially optimize the overall performance of the model, and our final MDL-IIA achieved the best overall performance (as shown in Table 3, MDL-IIA had an MCC of 0.837, outperforming other cohort models, all *p* values < 0.001). Based upon the t-SNE visualization, as shown in Fig. 4, the separation between the different classes improves by the addition of the designed modules, and more cancers are assigned to the correct molecular subtype categories by using the proposed model.

**Table 3:** Results of ablation tests for the proposed model in predicting 4-category molecular subtypes of breast cancer in the test cohort (*n* = 672).

Method	Modality	Accuracy (%)	Precision (%)	Recall (%)	F1-score	MCC
Multi-ResNet50	MG + US	84.4 [81.5, 87.1]	83.7 [80.4, 86.9]	81.3 [77.8, 84.5]	0.820 [0.786, 0.852]	0.777 [0.736, 0.814]
MulR-interSA	MG + US	85.4 [82.6, 88.1]	84.7 [81.5, 87.8]	82.3 [79.1, 85.6]	0.830 [0.797, 0.862]	0.793 [0.753, 0.829]
MulR-iiSA	MG + US	86.1 [83.6, 88.7]	86.1 [83.1, 88.9]	83.0 [79.8, 86.2]	0.839 [0.805, 0.870]	0.803 [0.767, 0.839]
MulR-interCSA	MG + US	87.5 [85.0, 90.0]	87.2 [84.3, 89.9]	84.6 [81.5, 87.7]	0.853 [0.823, 0.884]	0.822 [0.786, 0.858]
Proposed (MDL-IIA)	MG + US	88.5 [86.0, 90.9]	87.8 [85.0, 90.7]	85.4 [82.2, 88.4]	0.862 [0.831, 0.892]	0.837 [0.803, 0.870]

Values in brackets are 95% confidence intervals [95% CI, %].

*MG* mammography, *US* ultrasound, *MulR* Multi-ResNet, *SA* self-attention, *iiSA* intra- and inter-self-attention, *CSA* channel and spatial attention, *interSA* inter self-attention, *interCSA* inter channel and spatial attention, *MCC* matthews correlation coefficient, *MDL-IIA* multi-modal deep learning with intra- and inter-modality attention modules.

### Explainable AI

**Fig. 5:**
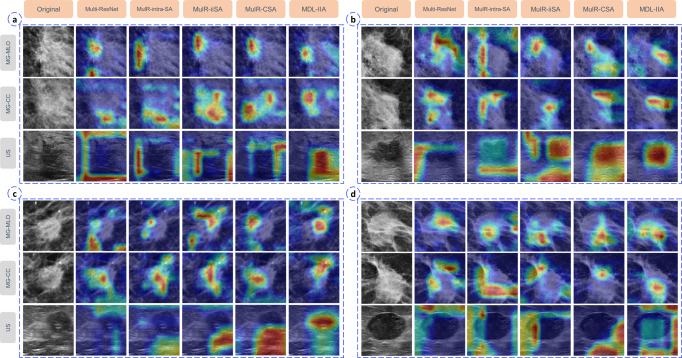
The visualization based on Gradient-weighted Class Activation Mapping (Grad-CAM) method of the proposed model in predicting 4-category molecular subtypes of breast cancer. Case **a**–**d** indicate the category of Luminal A, Luminal B, HER2-enriched and Triple-negative, respectively. MG mammography, CC craniocaudal, MLO mediolateral oblique, US ultrasound, MulR Multi-ResNet, SA self-attention, iiSA intra- and inter-self-attention, CSA channel and spatial attention, interSA inter self-attention, interCSA inter channel and spatial attention, MDL-IIA multi-modal deep learning with intra- and inter-modality attention modules.

In addition, multi-modal Gradient Weighted Class Activation Mapping (Grad-CAM) method was used to generate multi-modal heatmaps to visually explain MDL-IIA model decisions. Examples of visual heatmaps are shown in Fig. 5. The yellow and red positions are the regions of interest mostly used by the model for predicting the molecular subtype of breast cancer. The heatmaps are then analyzed to create understanding of image regions of particular interest to the model. We can observe that the addition of designed modules can reduce redundant or erroneous feature information from images, thereby enforcing the network to focus on the specific subtype. For mammography, we can observe that the proposed model focuses on the spiculation for Luminal A, the irregular shape and poorly defined margin for Luminal B, the calcification for HER2-enriched, and the regular shape for TN. For ultrasound, we can observe that the proposed model focuses on the irregular shape and shadow for Luminal A, the poorly defined margin and irregular shape for Luminal B, the poorly defined margin and calcification for HER2-enriched, and the information in/around the lesion, well defined margin and posterior echo for TN. These confirm that MDL-IIA can integrate features from different modalities and focus on the most predictive features of each molecular subtype of breast cancer.

### Discrimination of Luminal disease from Non-Luminal disease

**Fig. 6:**
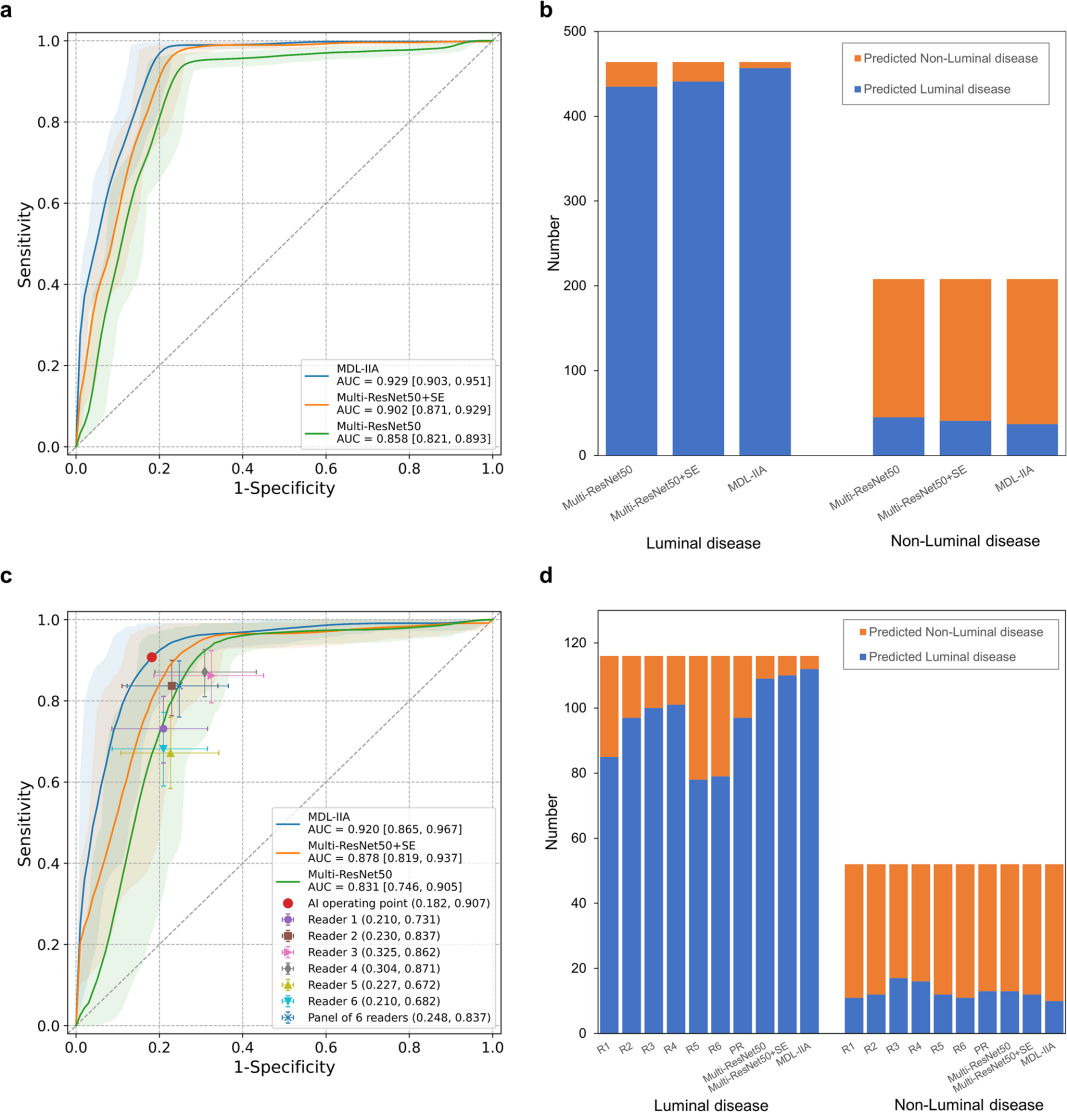
Performance of the proposed MDL-IIA model and radiologists. **a** The receiver operating characteristic (ROC) curve for distinguishing between Luminal disease and Non-Luminal disease by the proposed MDL-IIA model in the test cohort (*n* = 672). **b** The classification performance of the proposed MDL-IIA model in the test cohort (*n* = 672). **c** The ROC curve for distinguishing between Luminal disease and Non-Luminal disease by the proposed MDL-IIA model and the operating points of six radiologists in the observer study cohort (*n* = 168). **d** The classification performance of the proposed MDL-IIA model and six radiologists in the observer study cohort (*n* = 168). The 95% confidence intervals are shown as a shaded area for the ROC curve. MDL-IIA, multi-modal deep learning with intra- and inter-modality attention modules. Multi-ResNet50, multi-modal ResNet50 model. SE Squeeze-and-Excitation, PR panel of 6 readers, AI artificial intelligence, AUC area under the ROC curve.

A clinically important subtask, Luminal disease *vs* Non-Luminal disease (ER+ vs ER−), was considered for further analysis of the classification performance of the proposed model as this defines whether or not a cancer will be susceptible to endocrine therapy. In this setting, the MDL-IIA model needs to perform a binary classification to separate the Luminal disease group (Luminal A and Luminal B) from the Non-Luminal disease group (HER2-enriched and TN). As shown in Fig. 6a, the area under the receiver operating characteristic curve (AUC) was 0.929 (95% CI: 0.903, 0.951) for distinguishing between Luminal disease and Non-Luminal disease using MDL-IIA (Multi-ResNet50 with intra- and inter-modality attention modules) in the test cohort (*n* = 672), outperforming the model without attention mechanism (Multi-ResNet50, AUC of 0.858 (95% CI: 0.821, 0.893), *p* value < 0.001) and the model with SE attention mechanism (Multi-ResNet50+SE, AUC of 0.902 (95% CI: 0.871, 0.929), *p* value < 0.001). The MDL-IIA had an accuracy of 93.4% (95% CI: 91.5%, 95.2%), sensitivity of 98.5% (95% CI: 97.3%, 99.6%), specificity of 82.2% (95% CI: 76.9%, 87.4%), positive predictive value (PPV) of 92.5% (95% CI: 90.1%, 94.7%), and negative predictive value (NPV) of 96.1% (95% CI: 92.9%, 98.8%), adopting Non-Luminal disease as negative reference standard.

### Observer study

An observer study (*n* = 168, randomly selected 25% of the test cohort) was conducted to compare the performance between radiologists and AI model. In the task of identifying 4-category molecular subtypes of breast cancer, the accuracy and MCC of radiologists ranged from 56.5% to 68.0% and 0.428 to 0.568, respectively. And the accuracy and MCC was improved to 72.6% and 0.630 by the panel of 6 readers through majority vote, respectively. In contrast, MDL-IIA obtained higher accuracy (84.4%, *p* value < 0.001) and MCC (0.780, *p* value < 0.001) than radiologists (Supplementary Table 1 shows the individual performance results. Supplementary Figs. 1, 2 show the confusion matrix for radiologists and AI model).

In the task of distinguishing between Luminal disease and the Non-Luminal disease of breast cancer, the accuracy of radiologists ranged from 70.3% to 81.7%. Through majority vote by the panel of 6 readers, radiologists achieved an accuracy of 81.1%, sensitivity of 83.7%, specificity of 75.2%, PPV of 88.3% and NPV of 67.3% (Supplementary Table 2 shows all performance results. Supplementary Figs. 3 and 4 show the confusion matrix for radiologists and AI model). Figure 6c, d shows the performance of AI and radiologists for distinguishing between Luminal and Non-Luminal breast cancer in the observer study cohort. In this cohort, the MDL-IIA (Multi-ResNet50 with intra- and inter-modality attention modules) model obtained an AUC of 0.920 (95% CI: 0.865, 0.967), outperforming the model without attention mechanism (Multi-ResNet50, AUC of 0.831 (95% CI: 0.746, 0.905), *p* value < 0.001) and the model with SE attention mechanism (Multi-ResNet50+SE, AUC of 0.878 (95% CI: 0.819, 0.937), *p* value < 0.001). The MDL-IIA achieved superior or similar sensitivity as radiologists at the same specificity operating points. MDL-IIA had a sensitivity of 0.965 [0.929, 0.992] and a specificity of 0.810 [0.696, 0.913], which compared favorably with the panel of 6 radiologists (sensitivity of 0.837 [0.760, 0.898], specificity of 0.752 [0.628, 0.870], p values < 0.001). See Supplementary Table 2 for detailed results.

## Discussion

In this study, we report a multi-modal deep learning scheme with intra- and inter-modality attention modules for predicting the molecular subtypes of breast cancer leveraging both mammography and ultrasound images. Our main contributions and findings can be summarized as follows: (1) Multiple views of mammography and ultrasound images were combined to classify the molecular subtypes of breast cancer using deep learning models; (2) as a non-invasive method, the proposed MDL-IIA model could be directly used to predict the molecular subtypes of breast cancer, with an high accuracy of 88.5% (95% CI: 86.0%, 90.9%) and MCC of 0.837 (95% CI: 0.803, 0.870); (3) the proposed MDL-IIA model can also discriminate Luminal from Non-Luminal breast cancer cases, with an AUC of 0.929 (95% CI: 0.903, 0.951); (4) beyond the molecular-level IHC test, our method could potentially bypass the inherent uncertainty for HER2 score 2+ cases based on gene-level ground truth; (5) a reader study is conducted shows the superiority of this AI approach over assessment by clinicians.

Accurately determining the molecular subtype of breast cancer is an important factor for the prognosis of breast cancer patients and can guide treatment options. Current medical practitioners usually use IHC tests on breast biopsy specimens to determine the molecular subtype of breast cancer, but this method has many disadvantages, such as the high test cost, the interreader variability of pathologists and, obviously, the need for a biopsy specimen. Furthermore, breast tumors are highly heterogeneous, and the results of tissue samples taken from a specific location in the breast tumor may not be representative of the entire tumor. In recent years, hematoxylin-eosin-stained breast cancer pathological images-based AI models have been used in studies related to receptor status of breast cancer to overcome pathologists’ interreader variability^29–31,40^. However, the pathological images are also from the biopsy alone and may, therefore, not be completely representative. To overcome these difficulties, the combination of breast imaging and artificial intelligence has been used to predict the molecular subtypes of breast cancer in some studies. Many machine learning-based methods have been developed to predict the molecular subtypes of breast cancer^32–35^. However, traditional machine learning methods require complex feature engineering and cannot automatically extract useful features from images^41^, thus limiting their applications. Deep learning has sparked interest in improving the quality of automatic breast image interpretation due to its remarkable advances in automatic extraction and analysis of medical imaging. Ha et al.^42^ (*n* = 216), Zhang et al.^43^ (*n* = 244) and Sun et al.^44^ (*n* = 266) respectively developed MRI-based deep learning models to predict the molecular subtypes of breast cancer, but their small data sets limit the performance of their models. In addition, due to high examination costs and some other disadvantages (such as limited scanner availability, need for the injection of a contrast agent and long waiting time, etc.), MRI images may not be available for every patient^36^. Jiang et al.^45^ developed a deep learning model based on ultrasound to predict the molecular subtypes of breast cancer. However, the data labels used in this study were only based on the results of IHC, which is not completely accurate in determining the status of HER2, especially in HER2 score 2+ cases^17,18,46^. This will lead to deviations in the prediction results, thereby affecting the performance of the model. In addition, all the above studies did not combine images of different modalities. In contrast, our study is based on a large data set (*n* = 3360), and for each case multiple views of mammography and ultrasound images were used, that are routinely, and virtually everywhere in the world, available for all breast cancer patients; for data labeling, the labeling of molecular subtypes was not only based on IHC results, but used the results of the genetic analysis (SISH, silver-enhanced in situ hybridization) of equivocal/borderline HER2 cases to ensure the correctness of the annotated data. Although recent studies have attempted to use multimodal ultrasound images to predict molecular subtypes^47^, ultrasound-based images alone cannot provide non-ultrasonic features from other imaging modalities (such as mammography). Our study is a deep learning-based study to predict the molecular subtypes of breast cancer using multi-modal image analysis, combining mammography and ultrasound. The results of multi-modal deep learning models improve upon single-modal models. In addition, some studies in recent years have shown that attention mechanisms can potentially improve model performance^48–51^. In this study, we specifically proposed the intra- and inter-modality attention modules to better integrate features of images from different modalities, further increasing the accuracy of the final result.

As shown in results, our proposed model MDL-IIA recognized Luminal A and Luminal B better than HER2-enriched and TN. On one hand, since AI is somewhat data-driven, this may be due to differences in sample size, as the sample size of HER2-enriched and TN subgroups are relatively small because of the lower frequency of these types of breast cancer in clinical reality. On the other hand, it’s possible that the appearance of lower grade (Luminal A/B) cancers have more consistent features, whereas HER2-enriched and TN have features that are less consistent. The combination of mammography and ultrasound images in this study improved the performance of the model, which reflects the importance of multimodal images because they can provide more modality-specific features for identifying cancer subtypes. Therefore, further incorporation of MRI in multimodal models may potentially improve the performance of the model in future studies. In the results of the observer study cohort, the proposed MDL-IIA model performed similarly or better than radiologists. Radiologists identified some Luminal cases as TN cases, and had overall more errors in determining the 4-category molecular subtypes. This might be partly due to the fact that in clinical decision making the molecular subtypes are purely based upon pathological evaluation and radiologists are not really trained in this distinction. The higher performance of the MDL-IIA implies that more information is present. Therefore, radiologists may need more training in this area to improve their performance and could potentially also learn from the model output. No Luminal cases were predicted as being TN using the proposed MDL-IIA model, which may potentially prevent the unjust withholding of adjuvant endocrine treatment^52^. Particularly in areas of the world in which IHC staining is not everywhere available this may be of great value. Overall, the proposed MDL-IIA model has an AUC of 0.920 (95% CI: 0.865, 0.967) for discriminating Luminal disease from Non-Luminal disease in the observer study. The proposed MDL-IIA model can potentially also provide further decision support in combination with histopathology to assist doctors in evaluating and treating breast cancer patients. Beyond molecular-level results from IHC testing of location-specific tissue samples, our method enables prediction directly from entire cancer lesions and bypasses the inherent uncertainty based on gene-level ground truth. Due to the inhomogeneity of cancers particularly results in which the output of the model and the pathological analysis are discrepant are of interest. Automated analysis of medical images could improve our understanding of the downstream impact of imaging features, and lead to new insights into representative and discriminative morphological features for breast cancer.

There are also some limitations in this study. First, this study is a retrospective study with inevitable missing values. Second, the sample size of HER2-enriched and TN subgroups are relatively small due to the lower frequency of these types of breast cancer in clinical reality. Third, this is a feasibility study of cases obtained from a single center. Subsequent studies will collect more cases from multiple centers to externally verify the performance of the model. Fourth, radiologists are not really trained in predicting molecular subtypes of breast cancer due to the fact that in clinical decision making the molecular subtypes are purely based upon pathological evaluation. However, pathological assessment is prone to inter-observer variability. Cases where the high performing models were discrepant may be due to cases on pathology that are borderline and possibly not 100% accurate. Utilizing the predictions of our model in clinical practice might potentially lead to re-evaluation in highly discrepant cases and could potentially provide a higher level of radiology-pathology concordance. Although the model’s predictions and visualized heatmaps are not 100% correct, the heatmap visualization can show whether the images-based prediction is truly based upon image features of the cancer or is due to signals that may be erroneously interpreted as relevant to the classification outside the region of interest. This may help to use the AI findings in a more robust and explainable manner. In addition, the data used in this study came from only one mammography system and one ultrasound system, so the model may need to be retrained for data from other systems. However, this can be mitigated and future studies may benefit from using our public methods through transfer learning. Eventually, the potential effect of the use of MDL-IIA extracted molecular subtypes on the choice of therapy needs to be assessed.

In conclusion, we have developed the MDL-IIA model, which can potentially be used to predict the molecular subtypes and discriminate Luminal disease from Non-Luminal disease of breast cancer, while being a completely non-invasive, cheap and widely available effective method. Multi-modal imaging shows better performance than single-modal imaging, and intra- and inter-modality attention modules are shown to further improve the performance of our multi-modal deep learning model. This supports the idea that combining multi-modal medical imaging may indeed provide relevant imaging biomarkers for predicting therapy response in breast cancer, thereby potentially guiding treatment selection for breast cancer patients.

## Methods

### Data collection

This study was approved by Institutional Review Board of the Netherlands Cancer Institute with a waiver of informed consent (registration number: IRBd21-058). We retrospectively collected 4162 paired images (MG and corresponding US) of women with breast cancer presenting at the Netherlands Cancer Institute from January 2010 to November 2019. The MG exams were acquired with a HOLOGIC Selenia Dimensions mammography system and consisted of two image views per breast, the medio-lateral oblique (MLO) view and the cranio-caudal (CC) view. The US exams were acquired with a HITACHI HI VISION 900 ultrasound system, and a high-frequency linear probe has been used for all examinations. All patients had biopsy-proven breast cancers. The images used are pre-biopsy images, and therefore these images do not have biopsy scars. First, 4513 paired patches of all lesion locations were obtained according to the label marked by the dedicated breast radiologists. In this process, the size of the box for collecting lesions is adjusted to the size of the lesions, and included always surrounding normal tissue, as determined by the dedicated breast radiologists. Then, specific cases were deleted, including those with lesions that were not depicted in the images, those who had received preoperative intervention or treatment, and those without IHC results and SISH analysis. Finally, a total of 3360 paired cases were obtained, which were subdivided into 4 molecular subtypes according to the information of ER, PR, HER2 and Ki67 from IHC findings and SISH test (Supplementary Fig. 5 shows the definition and characteristics of molecular subtypes of breast cancer).

### Multi-modal deep learning model

The multi-modal deep learning algorithm was developed to predict the molecular subtypes of breast cancer. This model was combined with the attention mechanism to create the final model (multi-modal deep learning with intra- and inter-modality attention modules: MDL-IIA). Figure 2 shows the scheme for this study. For each case two views of mammography (MLO and CC) and an ultrasound image (showing the lesion) were used as input. First a baseline convolutional neural network was chosen, based upon the unadjusted performance of the classification task. The final model was designed based on the ResNet50 deep convolutional neural network and the attention mechanism, and was divided into four processing stages. As shown in Fig. 2, after the feature extractor of stage 1, the feature maps from MG-MLO and MG-CC were concatenated and then input to the attention module (stage 2, called intra-modality attention) to achieve intra-modality information interaction and obtain refined features. At the same time, the feature map from the US was also refined through the attention module. After the residual block of stage 3, the multi-modal feature maps are connected and then input to the multi-modal attention module (stage 4, called inter-modality attention) to realize the interaction of multi-modal information (Supplementary Table 3 shows the overall architecture of MDL-IIA, after the global average pooling layer, 2048 × 3 = 6144 features from the multi-modal image are extracted, and then analyzed through the fully connected layer to classify the cases).

In a convolutional neural network, although the receptive field will become larger as the depth of the network deepens, the convolution unit still only pays attention to local information each time, thus ignoring the impact of other global regions on the current region. Self-Attention was originally applied to natural language processing to capture the relationship between contexts, and has been applied to computer vision in recent years^53^. Therefore, self-attention was introduced to obtain the weight of the relationship between any pixel in the image and the current pixel. In the intra- and inter-modality attention modules, the input feature maps go through the self-attention mechanism to capture the spatial dependence of any two positions in the feature map to achieve the interaction of long-range features. As shown in Fig. 2a, the structure of self-attention was divided into three branches, called query (Q), key (K) and value (V). To reduce the amount of calculation, the number of channels of the feature map was first reduced by 1×1 convolution, where the number of filters for Q and K was reduced to one-eighth of the number of channels in the previous stage, while the number of filters for V was the same as the number of channels in the previous stage. Thereafter, the Q and each K were calculated through the similarity of the dot product to obtain the weights, and then the *softmax* function was used to normalize these weights to obtain the attention map. Finally, the weights and V were weighted and summed to obtain refined features. Inter-channel and spatial attention followed the inter-self-attention to realize the entire inter-modality attention module (Fig. 2b), the refined feature map by innter-channel attention (*RF*_*CA*_) is shown in Eq. (1).

1 $$RF_{CA} = {{{\mathrm{F}}}} \cdot \left( {\sigma \left( {MLP\left( {AvgPool\left( F \right)} \right) + MLP\left( {MaxPool\left( F \right)} \right)} \right)} \right)$$

and the refined feature map by inter-channel and spatial attention (*RF*_*CA*_) is shown in Eq. (2).

2 $$RF_{CSA} = RF_{CA} \cdot \left( {\sigma \left( {f^{3 \times 3}\left( {\left[ {AvgPool\left( {RF_{CA}} \right);MaxPool\left( {RF_{CA}} \right)} \right]} \right)} \right)} \right)$$

Where F represents the feature map for input, σ denotes the sigmoid function, MLP represents the shared multi-layer perceptron, *AvgPool* represents an average pooling operation, *MaxPool* represents a maximum pooling operation, *f*^3×3^ represents a convolution operation with the filter size of 3×3.

Ablation experiments were conducted to verify the effectiveness of the intra-modality and inter-modality attention modules in improving the classification performance. In detail, several network structures were chosen and compared to the proposed MDL-IIA model as follows: multi-modal ResNet50 model (called Multi-ResNet50), multi-ResNet with inter-modality self-attention module (called MulR-interSA), multi-ResNet with intra- and inter- self-attention modules (called MulR-iiSA), multi-ResNet with inter- channel and spatial attention modules (called MulR-interCSA).

All images were resized to 256 × 256 pixels and normalized (Supplementary Eqs. (1) and (2)) before model training. The initial parameters of the baseline model used the parameters pre-trained on ImageNet^54^, and all pre-trained parameters were trainable during the model training process. The MDL-IIA model was trained on NVIDIA RTX A6000 graphics processing unit (GPU), 48 Gigabytes of GPU memory. In the training cohort, the data was randomly divided into training set and validation set at a ratio of 4:1. In the training set, on-the-fly intensity and geometry augmentation was applied to avoid overfitting. The batch was set to 32 for 200 epochs and the initial learning rate was 1e-3 with a decay factor of 0.9 every 10 epochs. Adam optimizer was applied to update the model parameters (Supplementary Fig. 6 shows the F1 score and loss for training and validation set during training).

### Visualization: multi-modal Grad-CAM and t-SNE

To visually explain MDL-IIA model decisions, the multi-modal Gradient-weighted Class Activation Mapping (Grad-CAM) method based on the work of Selvaraju et al.^55^ was used to generate multi-modal heatmaps for understanding the focus of proposed model on images. The gradient *y*^*c*^ of the score for class c with respect to feature maps $A_{(m)^k}$ was first computed. Then, global average pooling was applied to these gradients to obtain the importance weights $a_{(m)_k^c}$ for unit k, as shown in Eq. (3).

3 $$a_{(m)_k^c} = \frac{1}{z}\mathop {\sum}\limits_i {\mathop {\sum}\limits_j {\frac{{\partial y^c}}{{\partial A_{\left( m \right)_{ij}^k}}}} }$$

Where Z represents the number of pixels in the corresponding feature map, *A*_*ij*_ represents the pixel value at position (i, j) of the k-th feature map, and m represents different image views or modalities, m = 1 refers to MG-MLO, m = 2 refers to MG-CC, and m = 3 refers to US. Finally, these weights were combined with the activation maps $( {A_{(m)_{ij}^k}})$, and then followed by a ReLU to obtain multi-modal gradient-weighted class activation mapping, as shown in Eq. (4).

4 $$L_{(m)_{Grad - CAM}^c} = {Re} LU\left( {\mathop {\sum}\limits_k {a_{\left( m \right)_k^c}A_{\left( m \right)^k}} } \right)$$

In addition, the non-linear dimensionality reduction method t-distributed stochastic neighborhood embedding (t-SNE) was used to visualize data related to breast cancer molecular subtypes in a two-dimensional space^56^. The t-SNE was applied to the flatten layer of the trained model.

### Observer study

In this study, a quarter of the test cohort cases were randomly selected as the observer study cohort. We aimed to measure the performance of radiologists in evaluating multi-modal images (MG and corresponding US) to distinguish the molecular subtypes and Luminal versus Non-Luminal breast cancers. As shown in Table 1, the reader study cohort contained a total of 168 cases, including 67 cases of Luminal A, 49 cases of Luminal B, 23 cases of HER2-enriched, and 29 cases of TN breast cancer. Six radiologists (L. A., R. M. M., J. V., R. W., K. M. D., and C. L.) with an average of 13 years (Supplementary Table 4 shows the individual experience level) of clinical experience analyzed the MG and US images (present at the same time) of each case to determine the molecular subtype of breast cancer that the case belongs to. The radiologists read cases without time limit and were blinded to the identity and medical background of the patients. Final decisions were made by a majority vote of a panel of 6 readers. When cases had equal votes, the decision of the more experienced group was followed.

### Statistical analysis

Statistical analysis was performed by SPSS (version 27.0) and Python 3.7, SPSS for data review and Python for data analysis. Packages used in Python include *numpy 1.19.2*, *pandas 1.2.4* and *scikit-learn 0.24.2*, etc. Check out the provided Github for more information. The accuracy, precision, recall, F1 score and Matthews correlation coefficient (MCC, ranged from -1 to 1, larger value means better performance) were used as evaluation indicators for predicting 4-category molecular subtypes. The confusion matrix was also used to evaluate prediction performance. AUC, accuracy, sensitivity, specificity, PPV and NPV were used as figures of merit to evaluate the performance of models and radiologists for distinguishing between Luminal disease and Non-Luminal disease. All calculation methods are shown in Eqs. (5)–(14). The operating point of AI model for distinguishing between Luminal disease and Non-Luminal disease was generated based on the maximum Youden index. 95% confidence intervals were generated with bootstrap method with 1000 replications^57^. The characteristics difference of the training and testing cohorts were compared by t-test or Mann–Whitney U test. T-test was used to compare the difference of indicators among different methods. All statistical analyses were two-sided and *p* value less than 0.05 was considered statistically significant.

All calculation methods are as follows:

5 $$Accuracy = \frac{1}{n}\mathop {\sum}\limits_{i = 1}^n {\frac{{TP_i + TN_i}}{{TP_i + TN_i + FP_i + FN_i}}}$$

6 $$Precision = \frac{1}{n}\mathop {\sum}\limits_{i = 1}^n {\frac{{TP_i}}{{TP_i + FP_i}}}$$

7 $$Recall = \frac{1}{n}\mathop {\sum}\limits_{i = 1}^n {\frac{{TP_i}}{{TP_i + FN_i}}}$$

8 $$F1\;score = \frac{1}{n}\mathop {\sum}\limits_{i = 1}^n {\frac{{TP_i}}{{TP_i + \frac{1}{2}\left( {FP_i + FN_i} \right)}}}$$

where *n* represents the number of categories.

9 $$MCC = \frac{{cov\left( {y\_true,\;y\_pred} \right)}}{{\sqrt {cov\left( {y\_true,\;y\_true} \right) \ast cov\left( {y\_pred,\;y\_pred} \right)} }}$$

where *y_true* is the ground truth target values, *y_pred* is the estimated targets as returned by the classifier.

10 $$Sensitivity = \frac{{TP}}{{TP + FN}}$$

11 $$Specificity = \frac{{TN}}{{TN + FP}}$$

12 $$PPV = \frac{{TP}}{{TP + FP}}$$

13 $$NPV = \frac{{TN}}{{TN + FN}}$$

where *TP* is true positive, *TN* is true negative, *FP* is false positive and *FN* is false negative.

14 $$AUC = \frac{\mathop {\sum}\nolimits_{ins_i \in positiveclass} {rank_{ins_i} - {\frac{{M \ast \left( {M + 1} \right)}}{2}}}}{M \ast N}$$

where M, N are the number of positive samples and negative samples respectively. $rank_{ins_i}$is the serial number of sample *i*. $\Sigma _{ins_i \in positiveclass}$ means add up the serial numbers of the positive samples.

### Reporting summary

Further information on research design is available in the Nature Research Reporting Summary linked to this article.

## Supplementary information

Supplementary Information

Reporting Summary

## Data Availability

The original annotation data is private and is not publicly available to guarantee protection of patients’ privacy. All data supporting the findings can be provided upon reasonable request to the corresponding author for non-commercial and academic purposes. Excel files containing raw data included in the main figures and tables can be found in the Source Data File in the article. We further provided all source codes of this study to facilitate the reproducibility.

## Source data

Source Data File

## Code availability

The codes of this paper are available at https://github.com/Netherlands-Cancer-Institute/Multimodal_attention_DeepLearning. It should only be used for academic research.
