# Supplementary material for: Predicting breast cancer types on and beyond molecular level in a multi-modal fashion
Source: NPJ Breast Cancer. 2023 Mar 22;9:16. doi: 10.1038/s41523-023-00517-2 (PMC10033710; doi:10.1038/s41523-023-00517-2)
Supplement: Supplementary file 1 — Supplementary Information [file 41523_2023_517_MOESM1_ESM.pdf]

Supplementary information

Predicting breast cancer types on and beyond molecular level in a multi-modal fashion

Tianyu Zhang et al.

Contents

I Supplementary Methods ... 2

Supplementary Methods 1. Normalization ... 2

II Supplementary Figures ... 3

Supplementary Figure 1. Performance of six radiologists for predicting 4-category molecular subtypes of breast cancer ... 3

Supplementary Figure 2. Performance of radiologists and AI for predicting 4-category molecular subtypes of breast cancer ... 4

Supplementary Figure 3. Performance of six radiologists for distinguishing between Luminal disease and Non-Luminal disease ... 5

Supplementary Figure 4. Performance of radiologists and AI for distinguishing between Luminal disease and Non-Luminal disease ... 6

Supplementary Figure 5. Definition and characteristics of molecular subtypes of breast cancer ... 7

Supplementary Figure 6. The F1 score and loss for training cohort ... 8

III Supplementary Tables ... 9

Supplementary Table 1. Performance of radiologists and AI in for predicting 4-category molecular subtypes of breast cancer in the observer study cohort ... 9

Supplementary Table 2. Performance of radiologists and AI for distinguishing between Luminal and Non-Luminal breast cancer in the observer study cohort ... 10

Supplementary Table 3. The overall architecture of the proposed model ... 11

Supplementary Table 4. Experience levels of the six radiologists ... 12

I Supplementary Methods

Supplementary Methods 1. Normalization

The normalization operation is a very important operation before the data is input into the deep learning model. As the raw input data values are distributed over different orders of magnitude and if normalization is not performed, some useful numerical features will be ignored, affecting the results of data analysis. Normalization is to limit the data to a certain range after certain processing. In this study, the image data of mammography and ultrasound were normalized fall into the range of 0-1 by normalization operation, and the normalization formula used was x = (x-min)/(max-min), where x represents the current pixel value, min and max represent the minimum and maximum values of pixels in the data set, respectively. It is worth noting that mammography (Equation 1) and ultrasound (Equation 2) were normalized separately since their corresponding data values are distributed on different orders of magnitude.

$$x\_mam = (x\_mam - min\_mam)/(max\_mam - min\_mam) \qquad (1)$$

$$x\_us = (x\_us - min\_us)/(max\_us - min\_us) \qquad (2)$$

II Supplementary Figures

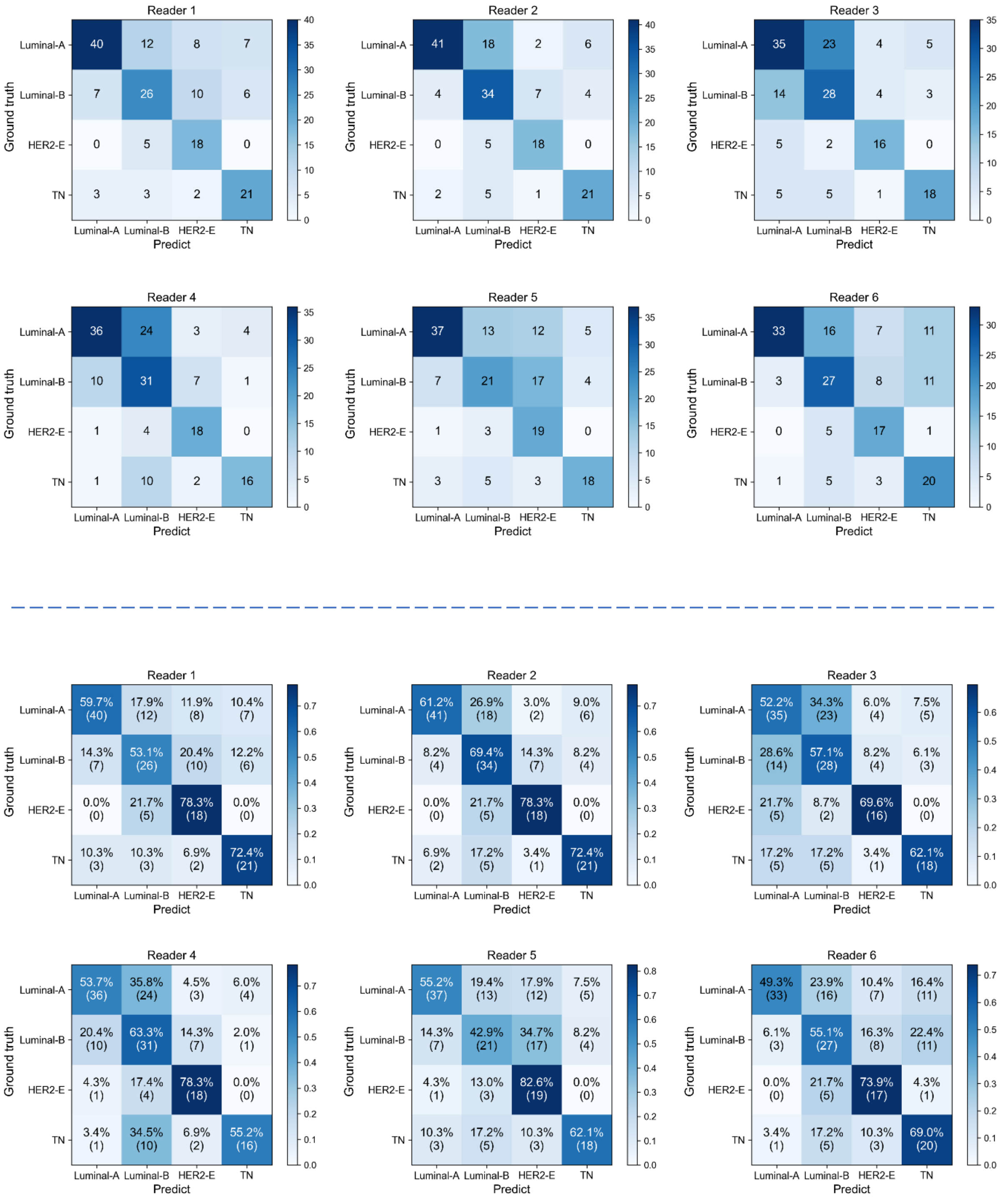

Supplementary Figure 1. Performance of six radiologists for predicting 4-category molecular subtypes of breast cancer.

Upper, original confusion matrix. Lower, normalized confusion matrix.

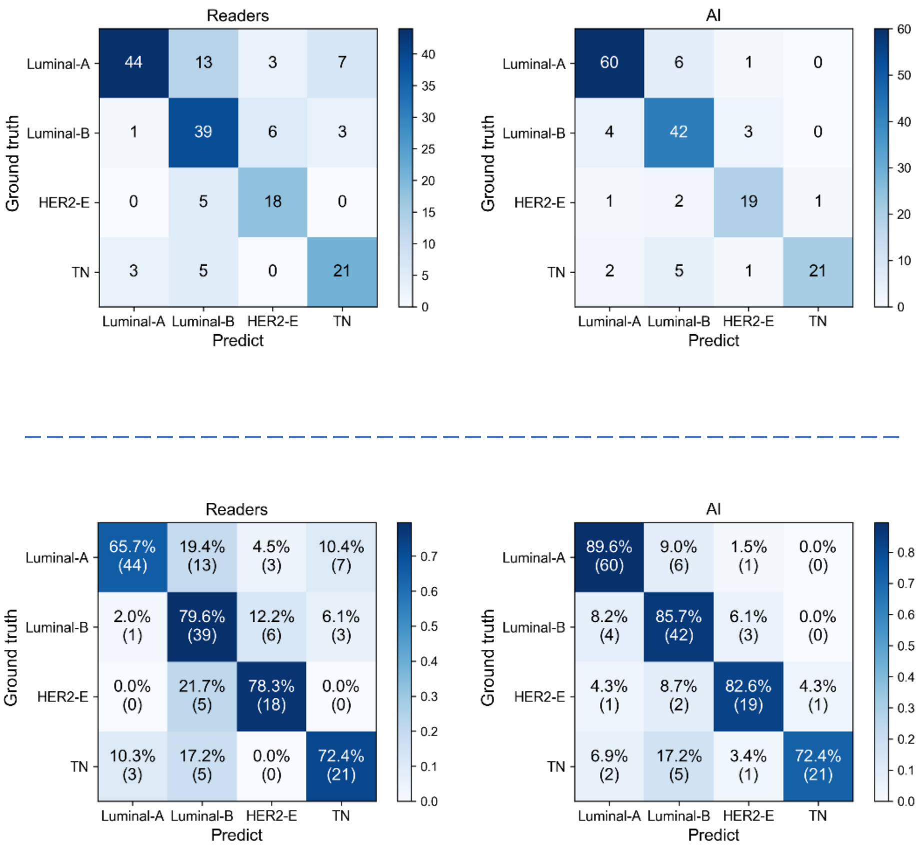

Supplementary Figure 2. Performance of radiologists (by the panel of 6 readers through majority vote) and AI for predicting 4-category molecular subtypes of breast cancer. Upper, original confusion matrix. Lower, normalized confusion matrix.

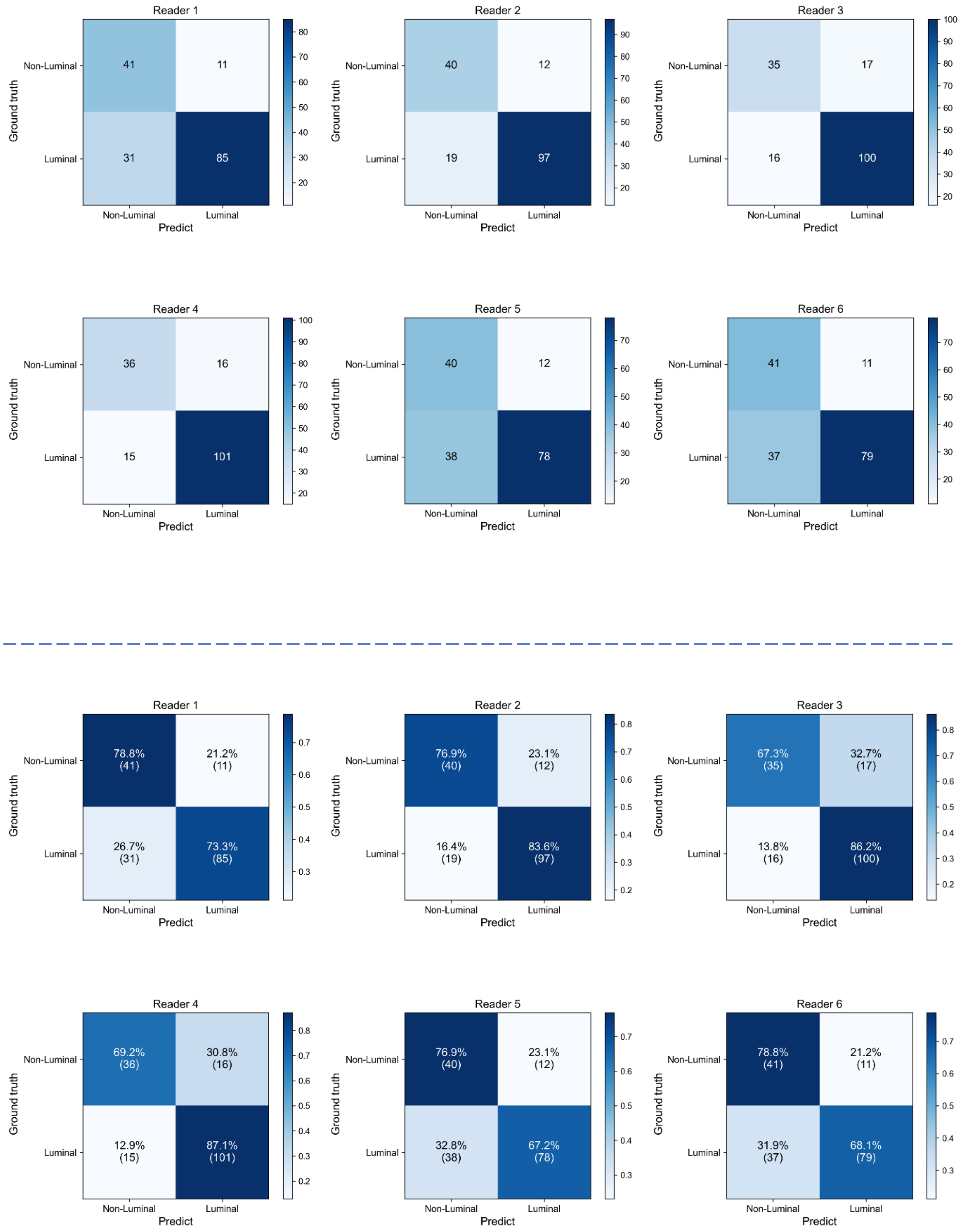

Supplementary Figure 3. Performance of six radiologists for distinguishing between Luminal disease and Non-Luminal disease. Upper, original confusion matrix. Lower, normalized confusion matrix.

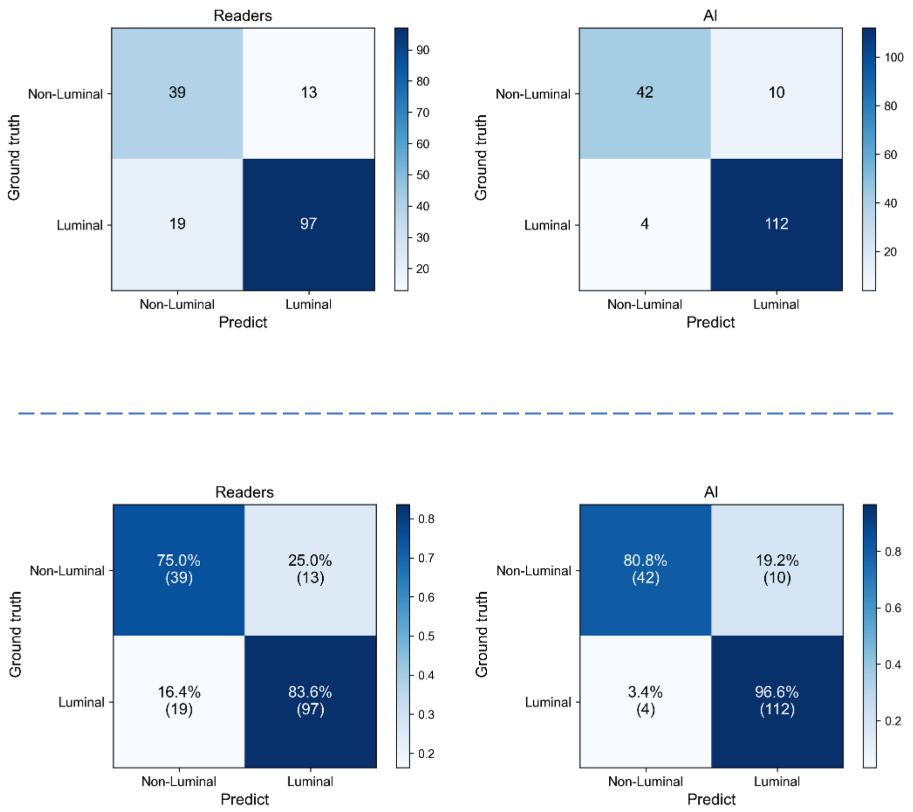

Supplementary Figure 4. Performance of radiologists (by the panel of 6 readers through majority vote) and AI for distinguishing between Luminal disease and Non-Luminal disease. Upper, original confusion matrix. Lower, normalized confusion matrix.

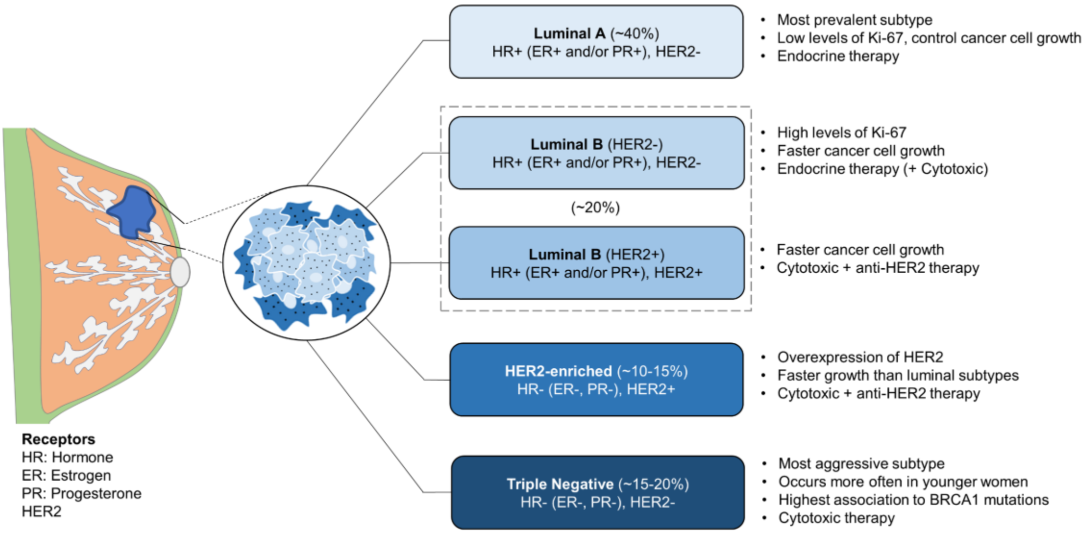

Supplementary Figure 5. Definition and characteristics of molecular subtypes of breast cancer. HER2, human epidermal growth factor receptor 2.

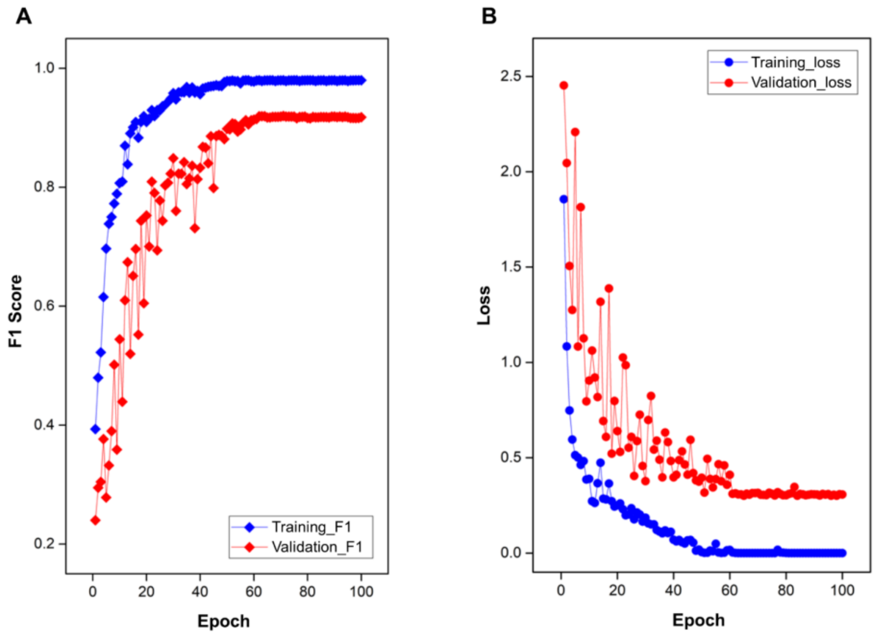

Supplementary Figure 6. The F1 score and loss for training cohort (training and validation set) of the proposed model in predicting molecular subtypes of breast cancer.

III Supplementary Tables

Supplementary Table 1. Performance of radiologists and AI in for predicting 4-category molecular subtypes of breast cancer in the observer study cohort.

Reader	Accuracy (%)	Precision (%)	Recall (%)	F1-score	MCC
Reader 1	62.4 [55.4, 69.6]	61.4 [54.0, 68.7]	65.9 [58.3, 73.0]	0.618 [0.545, 0.696]	0.496 [0.402, 0.596]
Reader 2	68.0 [60.7, 75.0]	68.7 [61.2, 75.9]	70.5 [62.5, 77.3]	0.683 [0.607, 0.749]	0.568 [0.468, 0.659]
Reader 3	57.7 [50.0, 64.9]	60.3 [52.1, 68.0]	60.3 [52.2, 68.3]	0.597 [0.517, 0.670]	0.408 [0.296, 0.515]
Reader 4	60.1 [52.3, 67.3]	64.0 [56.2, 71.1]	62.7 [54.7, 70.2]	0.615 [0.537, 0.685]	0.455 [0.346, 0.556]
Reader 5	56.5 [48.8, 64.3]	57.8 [50.0, 65.2]	60.8 [53.3, 68.0]	0.563 [0.485, 0.639]	0.428 [0.332, 0.528]
Reader 6	57.8 [50.0, 64.9]	58.8 [51.4, 65.6]	61.9 [53.8, 69.3]	0.573 [0.493, 0.650]	0.448 [0.353, 0.541]
Panel of 6 readers	72.6 [66.1, 79.2]	72.2 [65.3, 79.0]	74.0 [66.7, 80.7]	0.719 [0.649, 0.786]	0.630 [0.540, 0.717]
Proposed (MDL-IIA)	84.4 [78.6, 89.9]	85.0 [79.1, 90.8]	82.5 [75.8, 88.6]	0.831 [0.767, 0.893]	0.780 [0.703, 0.859]

Note: Values in brackets are 95% confidence intervals [95%CI, %]. MDL-IIA, multi-modal deep learning with intra- and inter-modality attention modules. MCC, matthews correlation coefficient.

Supplementary Table 2. Performance of radiologists and AI for distinguishing between Luminal and Non-Luminal breast cancer in the observer study cohort.

Reader	Accuracy (%)	Sensitivity (%)	Specificity (%)	PPV (%)	NPV (%)
Reader 1	74.9 [67.9, 81.5]	73.1 [64.7, 81.1]	79.0 [66.7, 89.6]	88.6 [81.4, 94.7]	56.8 [45.9, 68.4]
Reader 2	81.6 [75.6, 86.9]	83.7 [76.3, 90.0]	77.0 [65.1, 88.1]	89.1 [82.9, 94.7]	67.9 [55.1, 79.1]
Reader 3	80.4 [73.8, 86.3]	86.2 [79.5, 92.4]	67.5 [53.7, 80.0]	85.6 [78.6, 91.9]	68.7 [55.8, 81.4]
Reader 4	81.7 [76.2, 86.9]	87.1 [81.0, 92.7]	69.6 [57.6, 82.0]	86.5 [80.0, 92.4]	70.7 [58.1, 82.2]
Reader 5	70.3 [63.7, 76.8]	67.2 [58.4, 75.9]	77.3 [65.3, 88.9]	86.9 [80.0, 93.5]	51.3 [40.6, 62.4]
Reader 6	71.5 [64.3, 78.6]	68.2 [59.0, 77.2]	79.0 [66.7, 89.6]	87.9 [80.7, 94.4]	52.6 [41.7, 64.3]
Panel of 6 readers	81.1 [75.0, 86.3]	83.7 [76.0, 89.8]	75.2 [62.8, 87.0]	88.3 [81.9, 94.1]	67.3 [54.5, 78.9]
Ultrasound	85.1 [79.8, 90.5]	92.1 [86.6, 96.8]	69.5 [55.5, 81.6]	87.2 [80.8, 92.7]	79.6 [67.4, 91.5]
Multi-ResNet50	88.0 [82.7, 92.9]	93.8 [88.7, 97.7]	75.1 [61.4, 87.0]	89.5 [83.9, 94.8]	84.3 [72.7, 94.1]
Multi-ResNet50+SE	89.2 [83.9, 94.0]	94.7 [90.1, 98.3]	77.0 [63.8, 88.7]	90.3 [84.6, 95.3]	86.5 [75.9, 95.7]
Proposed (MDL-IIA)	91.7 [87.5, 95.8]	96.5 [92.9, 99.2]	81.0 [69.6, 91.3]	91.9 [86.4, 96.7]	91.1 [82.2, 97.9]

Note: Values in brackets are 95% confidence intervals [95%CI, %]. MDL-IIA, multi-modal deep learning with intra- and inter-modality attention modules. SE, Squeeze-and-Excitation. PPV, positive predictive value. NPV, negative predictive value.

Supplementary Table 3. The overall architecture of the proposed model.

<table>
<tr><th>Modality</th><th>MG-MLO</th><th>MG-CC</th><th>US</th></tr>
<tr><td>Input 1 size</td><td>256 × 256 × 1</td><td>256 × 256 × 1</td><td>256 × 256 × 1</td></tr>
<tr><td>Stage 1</td><td>$\begin{bmatrix} 7 \times 7, 64, stride\ 2 \\ 3 \times 3\ \max pool, stride\ 2 \end{bmatrix}$
$\begin{bmatrix} 1 \times 1, 64 \\ 3 \times 3, 64 \\ 1 \times 1, 256 \end{bmatrix} \times 3$
$\begin{bmatrix} 1 \times 1, 128 \\ 3 \times 3, 128 \\ 1 \times 1, 512 \end{bmatrix} \times 4$
$\begin{bmatrix} 1 \times 1, 256 \\ 3 \times 3, 256 \\ 1 \times 1, 1024 \end{bmatrix} \times 6$</td><td>$\begin{bmatrix} 7 \times 7, 64, stride\ 2 \\ 3 \times 3\ \max pool, stride\ 2 \end{bmatrix}$
$\begin{bmatrix} 1 \times 1, 64 \\ 3 \times 3, 64 \\ 1 \times 1, 256 \end{bmatrix} \times 3$
$\begin{bmatrix} 1 \times 1, 128 \\ 3 \times 3, 128 \\ 1 \times 1, 512 \end{bmatrix} \times 4$
$\begin{bmatrix} 1 \times 1, 256 \\ 3 \times 3, 256 \\ 1 \times 1, 1024 \end{bmatrix} \times 6$</td><td>$\begin{bmatrix} 7 \times 7, 64, stride\ 2 \\ 3 \times 3\ \max pool, stride\ 2 \end{bmatrix}$
$\begin{bmatrix} 1 \times 1, 64 \\ 3 \times 3, 64 \\ 1 \times 1, 256 \end{bmatrix} \times 3$
$\begin{bmatrix} 1 \times 1, 128 \\ 3 \times 3, 128 \\ 1 \times 1, 512 \end{bmatrix} \times 4$
$\begin{bmatrix} 1 \times 1, 256 \\ 3 \times 3, 256 \\ 1 \times 1, 1024 \end{bmatrix} \times 6$</td></tr>
<tr><td>Output 1 size</td><td>16 × 16 × 1024</td><td>16 × 16 × 1024</td><td>16 × 16 × 1024</td></tr>
<tr><td>Transition</td><td colspan="2">Concatenate (MG-MLO and MG-CC)</td><td>--</td></tr>
<tr><td>Input 2 size</td><td colspan="2">16 × 32 × 1024</td><td>16 × 16 × 1024</td></tr>
<tr><td>Stage 2 (Intra-Modality Attention)</td><td colspan="2">Intra-Self-Attention</td><td>Intra-Self-Attention</td></tr>
<tr><td rowspan="2">Input 3 (output 2) size</td><td colspan="2">16 × 32 × 1024</td><td rowspan="2">16 × 16 × 1024</td></tr>
<tr><td>16 × 16 × 1024</td><td>16 × 16 × 1024</td></tr>
<tr><td>Stage 3</td><td>$\begin{bmatrix} 1 \times 1, 512 \\ 3 \times 3, 512 \\ 1 \times 1, 2048 \end{bmatrix} \times 3$</td><td>$\begin{bmatrix} 1 \times 1, 512 \\ 3 \times 3, 512 \\ 1 \times 1, 2048 \end{bmatrix} \times 3$</td><td>$\begin{bmatrix} 1 \times 1, 512 \\ 3 \times 3, 512 \\ 1 \times 1, 2048 \end{bmatrix} \times 3$</td></tr>
<tr><td>Output 3 size</td><td>8 × 8 × 2048</td><td>8 × 8 × 2048</td><td>8 × 8 × 2048</td></tr>
<tr><td>Transition</td><td colspan="3">Concatenate (MG-MLO, MG-CC and US) (8 × 24 × 2048)</td></tr>
<tr><td rowspan="3">Stage 4 (Inter-Modality Attention)</td><td colspan="3">Inter-Self-Attention</td></tr>
<tr><td colspan="3">Reshape (8 × 8 × 6144)</td></tr>
<tr><td colspan="3">Inter-Channel-Spatial-Attention</td></tr>
<tr><td>Output 4 size</td><td colspan="3">(8 × 8 × 2048) × 3</td></tr>
<tr><td>GAP layer</td><td colspan="3">GAP layer</td></tr>
<tr><td>Output 5 size</td><td colspan="3">(1 × 1 × 2048) × 3</td></tr>
<tr><td>FC layer</td><td colspan="3">FC layer</td></tr>
<tr><td>Output</td><td colspan="3">Luminal A, Luminal B, HER2-enriched and Triple-negative</td></tr>
</table>

Note: MG, mammography. US, ultrasound. MLO, medio-lateral oblique. CC, cranio-caudal. GAP, Global Average Pooling. FC, Fully-connection.

Supplementary Table 4. Experience levels of the six radiologists involved in our reader study.

Reader	Years of experience
Reader 1	6
Reader 2	14
Reader 3	16
Reader 4	9
Reader 5	13
Reader 6	20
Average	13

Note: Years quoted are years practicing as dedicated breast radiologist. This means after medical school, general radiology training and either a fellowship or a PhD in breast imaging.
