# Supplementary material for: Predicting breast cancer types on and beyond molecular level in a multi-modal fashion
Source: NPJ Breast Cancer. 2023 Mar 22;9:16. doi: 10.1038/s41523-023-00517-2 (PMC10033710; doi:10.1038/s41523-023-00517-2)
Supplement: Supplementary file 2 — Reporting Summary [file 41523_2023_517_MOESM2_ESM.pdf]

nature portfolio

Corresponding author(s): Tao Tan

Last updated by author(s): May 23, 2022

Reporting Summary

Nature Portfolio wishes to improve the reproducibility of the work that we publish. This form provides structure for consistency and transparency in reporting. For further information on Nature Portfolio policies, see our Editorial Policies and the Editorial Policy Checklist.

Statistics

For all statistical analyses, confirm that the following items are present in the figure legend, table legend, main text, or Methods section.

n/a	Confirmed	
☐	☒	The exact sample size (n) for each experimental group/condition, given as a discrete number and unit of measurement
☐	☒	A statement on whether measurements were taken from distinct samples or whether the same sample was measured repeatedly
☐	☒	The statistical test(s) used AND whether they are one- or two-sided<br>*Only common tests should be described solely by name; describe more complex techniques in the Methods section.*
☒	☐	A description of all covariates tested
☒	☐	A description of any assumptions or corrections, such as tests of normality and adjustment for multiple comparisons
☐	☒	A full description of the statistical parameters including central tendency (e.g. means) or other basic estimates (e.g. regression coefficient) AND variation (e.g. standard deviation) or associated estimates of uncertainty (e.g. confidence intervals)
☐	☒	For null hypothesis testing, the test statistic (e.g. F, t, r) with confidence intervals, effect sizes, degrees of freedom and P value noted<br>*Give P values as exact values whenever suitable.*
☒	☐	For Bayesian analysis, information on the choice of priors and Markov chain Monte Carlo settings
☐	☒	For hierarchical and complex designs, identification of the appropriate level for tests and full reporting of outcomes
☒	☐	Estimates of effect sizes (e.g. Cohen's d, Pearson's r), indicating how they were calculated

Our web collection on statistics for biologists contains articles on many of the points above.

Software and code

Policy information about availability of computer code

Data collection	HITACHI HI VISION 900 ultrasound system. HOLOGIC Selenia Dimensions mammography system.
Data analysis	SPSS 27.0; Python 3.7.

For manuscripts utilizing custom algorithms or software that are central to the research but not yet described in published literature, software must be made available to editors and reviewers. We strongly encourage code deposition in a community repository (e.g. GitHub). See the Nature Portfolio guidelines for submitting code & software for further information.

Data

Policy information about availability of data

All manuscripts must include a data availability statement. This statement should provide the following information, where applicable:

- Accession codes, unique identifiers, or web links for publicly available datasets
- A description of any restrictions on data availability
- For clinical datasets or third party data, please ensure that the statement adheres to our policy

The original annotation data is private and is not publicly available to guarantee protection of patients' privacy. All data supporting the findings including the imaging data can be provided upon reasonable request to the corresponding author for non-commercial and academic purposes, and all requests will be promptly reviewed within 15 working days. We further provided all source codes of this study to facilitate the reproducibility.

Field-specific reporting

Please select the one below that is the best fit for your research. If you are not sure, read the appropriate sections before making your selection.

☒ Life sciences ☐ Behavioural & social sciences ☐ Ecological, evolutionary & environmental sciences

For a reference copy of the document with all sections, see nature.com/documents/nr-reporting-summary-flat.pdf

Life sciences study design

All studies must disclose on these points even when the disclosure is negative.

Sample size	The inclusion criteria included the followings: (a) women with breast cancer with paired mammography and ultrasound images; (b) availability of clinical data; (c) cases with immunohistochemistry results and silver-enhanced in situ hybridization analysis. Finally, 3360 paired cases were enrolled for analysis.
Data exclusions	Specific cases were deleted, including those with unclear lesions in the image, those who had received preoperative intervention or treatment, those without immunohistochemistry results and silver-enhanced in situ hybridization analysis.
Replication	Models were verified and replicated using regular machine learning metrics on independent test cohort.
Randomization	The enrolled samples were randomly divided into the training cohort and test cohort.
Blinding	The investigator were blinded to group allocation during data collection and analysis.

Reporting for specific materials, systems and methods

We require information from authors about some types of materials, experimental systems and methods used in many studies. Here, indicate whether each material, system or method listed is relevant to your study. If you are not sure if a list item applies to your research, read the appropriate section before selecting a response.

Materials & experimental systems

n/a	Involved in the study
☒	☐ Antibodies
☒	☐ Eukaryotic cell lines
☒	☐ Palaeontology and archaeology
☒	☐ Animals and other organisms
☒	☐ Human research participants
☐	☒ Clinical data
☒	☐ Dual use research of concern

Methods

n/a	Involved in the study
☒	☐ ChIP-seq
☒	☐ Flow cytometry
☒	☐ MRI-based neuroimaging

Clinical data

Policy information about clinical studies

All manuscripts should comply with the ICMJE guidelines for publication of clinical research and a completed CONSORT checklist must be included with all submissions.

Clinical trial registration	This study does not prospectively assign participants to any interventions and so is not a clinical trial which requires listing.
Study protocol	The study protocol is appended with the paper.
Data collection	Between January 2010 to November 2019, a total of 3360 paired cases (mammography and corresponding ultrasound) were obtained from the Netherlands Cancer Institute. Clinical and histopathologic data were obtained from the medical records.
Outcomes	We assess different methods by calculating the diagnostic performances in predicting 4-category molecular subtypes of breast cancer (Luminal A, Luminal B, HER2-enriched and Triple-negative), and distinguishing between Luminal disease and Non-Luminal disease breast cancers. A reader study is conducted to show the superiority of the proposed AI model over clinicians.
